# The lived experience of performing a periodontal treatment in the context of general dentistry

**DOI:** 10.1038/s41405-021-00059-4

**Published:** 2021-01-28

**Authors:** Aleksandar Milosavljevic, Eva Wolf, Magnus Englander, Andreas Stavropoulos, Bengt Götrick

**Affiliations:** 1 Department of Periodontology, Faculty of Odontology, Malmö University, SE-205 06 Malmö, Sweden; 2 Department of Endodontics, Faculty of Odontology, Malmö University, SE-205 06 Malmö, Sweden; 3 Department of Social Work, Faculty of Health and Society, Malmö University, SE-205 06 Malmö, Sweden; 4 Department of Oral Diagnostics, Faculty of Odontology, Malmö University, SE-205 06 Malmö, Sweden

**Keywords:** Periodontics, Periodontitis

## Abstract

**Aim:**

To describe what characterises the lived experience of performing a periodontal treatment in the context of general dentistry.

**Materials and methods:**

Three dental hygienists from general dentistry in Sweden, were purposively selected as participants and interviewed. The participants described a situation in which they had performed a periodontal treatment. The descriptions were analysed using the descriptive phenomenological psychological method.

**Results:**

The general meaning structure of the lived experience of performing a periodontal treatment comprised five constituents, (a) an established treatment routine, (b) importance of oral hygiene, (c) self-awareness and motivation of the patient, (d) support and doubt, and (e) mechanical infection control. The periodontal treatment is perceived as being set prior to its commencement and as following established routines, in which the patients’ oral hygiene is experienced as a crucial part. The patients’ self-awareness and a supportive clinician are seen as important factors in motivating the patient towards positive behavioural change, although there is a presence of doubt in patients’ ability to maintain this positive change. Mechanical infection control is perceived as successful but sometimes difficult to perform.

**Conclusions:**

Important, patient-related, factors are constituting the phenomenon of performing a periodontal treatment but an experience that the pre-existing standardised workflow influences patient management was also present.

## Introduction

Chronic periodontitis is a common and prevalent disease in the adult population,^1–3^ where the severe form affects almost 11% of the worldwide population. This means that severe periodontitis is the 6th most prevalent disease of mankind.^4^ The treatment of periodontal diseases is mainly focused on cause-related therapy which implies removal and control of plaque^5^ through mechanical tooth cleaning and scaling.^6,7^ Moreover, providing the patient with the motivation to maintain a sufficient oral hygiene routine is generally important,^8^ but it is also an essential factor in the success of scaling.^9^ Therefore, the key element in achieving gingival health is to set up an individually tailored approach, i.e. to individually tailor oral hygiene instructions to patients^10^ and enhance their motivation by increasing their knowledge of the disease as well as their understanding of the benefits resulting from changing their behaviour.^11^

Although an individually tailored treatment would be a preferable approach in the care of patients with periodontal diseases, such an approach is not always implemented. Some questionnaire studies have shown that both general dental practitioners and dental hygienists (DHs) in general dentistry do not always adapt the treatment to the treatment needs of the individual patient.^12,13^ Consequently, patients were assigned a similar treatment with respect to the amount of treatment time and treatment measures, instead of being adapted to the different periodontal conditions of the patients. In terms of a longer perspective, the standardised approach has been shown to be less successful than an individually tailored oral health educational programme that is more effective in reducing the amount of plaque and gingival bleeding.^14^ Furthermore, an individually tailored programme also improves the success rate of non-surgical treatment when used in combination with scaling,^15^ this combination also carries a low incremental cost per patient treated.^16^ Hence, it is important to clarify the working environment in Swedish general dentistry, in order to better understand why patients’ periodontal treatment is still, in most cases, not individually tailored. One way of doing this is to obtain a deeper understanding of how periodontal treatment is experienced in the general dentistry context, as seen from the professional perspective of DHs. The Swedish DHs are well-known to perform most of the non-surgical periodontal treatment, independently, in this particular setting. Furthermore, the non-surgical periodontal treatment performed by the DHs constitutes the largest part (>90%) of the total periodontal treatment a patient in the general dentistry setting receives.^17^ There are only a few studies which have considered the DHs’ own experience of periodontal treatment, although some researchers have made inquiries into the psychosocial process of DHs when striving toward a successful periodontal treatment,^18^ and the influence of DHs’ working environment on their job satisfaction.^19^ There are few studies that have dealt with the lived experience of performing dental treatment, e.g. when dental professionals manage children with carious lesions^20^ but no study has yet explored what it means to perform a periodontal treatment in the professional context of general dentistry.

The aim of this study was to make an in-depth qualitative study in which interviewing of only a few participants was utilised in order to capture the qualitative nuances in clinical work. There are currently many qualitative methods available, however, one method that has had a focus on how a particular phenomenon (in this study a lived experience of a clinical process) is lived through and also how such a phenomenon would be related to a context (in this study, the context of general dentistry) is the descriptive phenomenological psychological method (DPPM).^21^

## Materials and methods

The qualitative research method as selected for this study, DPPM, consists of three steps: (1) obtain a description from research participants of a situation in which the phenomenon was experienced, (2) adopt a reflective attitude in order to focus on the relation between the lived experience and the clinical process being studied, while at the same time keeping in mind the interdependency between the phenomenon and the overall context and (3) clarify and describe the essential, qualitative structure of the lived experience of the clinical process across all the three experiential situations selected.^21^

### Data collection

In contrast to selection criteria in a study focusing on making general knowledge claims on a specific population, a qualitative phenomenological study seeks what is general about a phenomenon, such as the lived experience of a clinical process.^22^ As Stewart et al.^23^ has shown in an earlier BDJ article explaining the differences between quantitative and qualitative inquiry, *‘Methods of data collection used in qualitative research differ from those used in quantitative research because of a fundamental epistemological difference which underpins qualitative and quantitative methodologies’*. Hence, there are fundamental differences between qualitative and quantitative studies. In this study, three clinically active DHs from both public and private general dentistry in the county of Skåne, Sweden, were selected, because, and following qualitative research criteria, they could provide rich descriptions of situations in which they had a lived experience of the phenomenon (i.e. the lived experience of a periodontal treatment). This group was purposively selected because they treat patients with periodontal disease on a daily basis. Hence, the inclusion criteria for the DHs were that they were currently performing non-surgical periodontal treatment on a regular basis, independently, and had a recent memory of a situation in which they had performed the treatment process. Moreover, the specific DHs were strategically selected as they, in addition to fulfilling the inclusion criteria, also had diverse demographic characteristics in terms of age, gender, professional experience in general dentistry, place of education and employer setting. The aim was to obtain descriptions from individuals with different backgrounds and professional experiences (Table 1). This sampling strategy was conducted so that the systematic focus remained on the phenomenon and its relation to a context.^24^ The participants were also selected purposively with as much demographic variety as possible in order not to confuse selection criteria with sampling strategies in population research and to keep a clear focus on the phenomenon and not on the individual.^22,24^

**Table 1:** Participants (dental hygienists) demographics.

Participant	Age	Gender	Professional experience^a^	Place of education	Employer
A	38	Female	13 Years	Malmö University	Public
B	35	Male	11 Years	Kristianstad University	Public
C	55	Female	31 Years	Malmö University	Private

^a^Continuous professional experience in a general dentistry setting.

Two separate meetings were conducted between the interviewer (A.M.) and each of the three participants. The interviewer was a 32-year-old male, general dentist with 8 years of professional experience, undergraduate educator as well as Ph.D. student in periodontology. Moreover, the interviewer was also experienced with interviewing in qualitative studies. During the first meeting, the participants were informed about the study aim, signed a consent form, and were asked to mentally review a situation where they had performed a periodontal treatment on a patient. The second meeting (in-depth semi-structured interview) was scheduled to approximately a week after the first meeting. They were also informed that the participation was confidential and voluntary with the right to discontinue participation at any time.

The second meeting was initiated with the following question to the participants: ‘please describe, in as much detail as possible, a situation where you performed a periodontal treatment on a patient’. The participants independently chose which situation to recall and describe. During the interview, the participants could freely describe the situations and the interviewer only asked for clarification and a more detailed description when this was needed. Three in-depth semi-structured interviews were conducted in the above manner. The interviews lasted between 38 and 64 min. The interviews were documented using a digital sound recorder and transcribed verbatim. The study was approved by the Regional Ethical Board at Lund University, Lund, Sweden (LU-752/2013).

### Data analysis

**Fig. 1:**
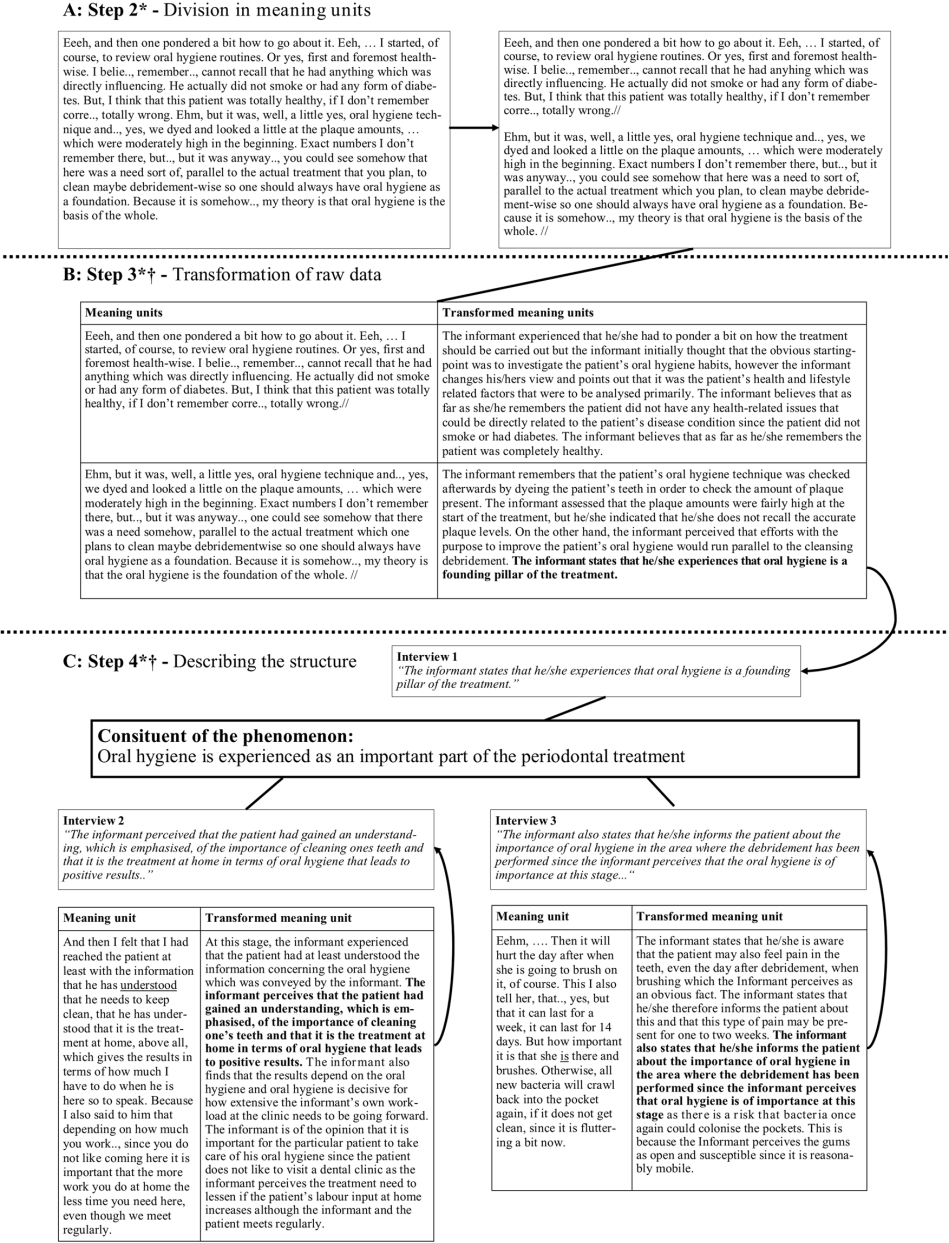
Simplified example of the last three methodological steps (steps 2–4) in the analysis of the descriptions. **A** Example of the process of transforming the interview in meaning units. **B** Example of how raw data is analysed and transformed. **C** Example of how a part of a general structure is created. *Phenomenological reduction applied in the step. ^†^Eidetic variations applied in the step.

The analysis of the interview material followed four consecutive methodological steps and was conducted by three researchers (A.M., E.W. and M.E.). Two of the researchers (A.M., E.W.), with experience in qualitative research and dentistry, analysed the whole material with input and feedback from a researcher with vast experience in DPPM (M.E.). In the first step, each transcribed interview was read several times in order to get a sense of the whole, that is, providing the researcher with the overall context and its relation to the lived experience of the clinical process. In the second step, the transcribed interview was divided into so-called meaning units, first and foremost a step, in order to render the analysis more manageable (Fig. 1A). In the third step, a repeated transformation of the raw data was made from each meaning unit. The purpose was to critically distinguish and describe the precise psychological meaning of each meaning unit and thus identify the phenomenon’s constituents (i.e. bearing elements). All data were accounted for which means that nothing was added or removed during the qualitative phenomenological analysis. In particular, the researcher focuses in this third step on the qualitative relation between the participants experience and the clinical process, as well as how the context provides the ground for the phenomenon. Technically in phenomenological inquiry, this focus is referred to as the researcher utilising the phenomenological method, which is a reflective attitude in order to provide for a specific focus on the phenomenon (in this study: how the participants experience relate to the clinical process) as it appears in an empirical context (in this study: the context of general dentistry). To guide this methodological process, the researcher asked the following question when approaching each meaning unit: ‘What does this particular meaning unit tell me about the lived experience of performing a periodontal treatment?’ The written transformation of each meaning unit was critically varied several times until the most precise answer to the above question was found. At the end of this step, a series of transformed meaning units for each interview was obtained (Fig. 1B). In the fourth step, all transformed meaning units from all three interviews were varied in order to articulate and describe the invariant qualitative structure of the phenomenon (Fig. 1C).

## Results

The results consist of a general qualitative structure of the phenomenon of ‘the lived experience of performing a periodontal treatment’ in the context of general dentistry, which in turn comprises five different constituents (i.e. bearing elements) and their interdependent relations. As in all phenomenological inquiry, a qualitative structure is always provisional (technically in phenomenology language: morphological) and should thus not be compared to structures in mathematics and logic that are rendered exact structures. This structure is based on an identification of the phenomenon’s constituents, which were present in all three descriptions and should be considered as a coherent whole on the limitation and basis of the data gathered.

### The general structure

The psychological meaning of performing a periodontal treatment in the context of general dentistry is characterised by an established treatment routine following a set pattern. The planning of the arrangement is defined by a requirement to base the treatment on the premises of the individual patient by attaining a holistic understanding. The extent of the entire treatment is based on the perception of deep periodontal pockets and their frequency whereas a single treatment session is instead influenced by the time available. In the situation where the phenomenon is experienced, patients are perceived as being only partially aware of their oral condition and somewhat mindful of the demands placed on their oral hygiene. Patients’ increased self-awareness is understood as an important factor in order to set up for the possibility of increased motivation for behavioural change in the direction of improving the patient’s oral hygiene. The perception of patients’ capacity to maintain a good oral hygiene is an integral part of the lived experience when conducting the periodontal treatment. The patients’ oral hygiene capacity is perceived to be a crucial factor in obtaining successful results. Instructions in technique and choice of oral hygiene appliances is perceived as playing a pivotal role in the treatment process as is the patients’ integration of instructions, techniques, and the use of appliances into their daily routines. The patients’ integration of this self-care action is experienced as necessary in order to achieve an improved oral hygiene. The patients are perceived to adhere to the instructions and retain their motivation, but at the same time there is a sense of doubt that such positive behaviour would actually manifest itself in the patients’ self-care process. Another fundamental characteristic of the phenomenon is the use of scaling that is considered successful although difficulties are experienced with regards to gaining visual and physical access to certain areas of the mouth. The combination of the use of ultrasonic and hand instruments is perceived as resulting in a thorough cleaning of the teeth surfaces. When carrying out the periodontal treatment, there is also an experience of a need to be supportive, in a professional way, of the patient.

### Constituent A: an established treatment routine

The periodontal treatment arrangement is experienced as being a previously established and familiar routine. It is for the most part performed routinely and follows a set pattern. The planning of the whole treatment is experienced as being based on information consisting of the patient’s general health status, daily routines, and oral hygiene routines.

- *I think that it is important that you familiarise yourself with the patient and that you not just like only look into the oral cavity and see that there are 32 teeth and so on, but that you get a.., a holistic perspective. Eeh, health wise.., well what their routines are, oral hygiene, diet and so on, those regular basic bits that we can diagnose through., that we need to have as a basis for our.. treatment course*.
  Furthermore, several factors are experienced as being important when planning a single treatment session: patient’s susceptibility to the information intended to be given, how much information the patient can receive, the present stage of the treatment, and the stress level of the patient.
- *Well, you do take your cue from the individual: how much information they can take on and where they are, if they are stressed or if they are susceptible to information*.
  In general, these factors were expressed explicitly but an implicit reflection of the patient’s daily life, habits, and oral hygiene routines was also present and formed the course of the treatment. Moreover, frequency and magnitude of deep periodontal pockets were experienced as vital when planning the extent of the treatment in terms of number of sessions.
- *So, my plan is sort of already there, when I measure pockets, that this is like.., this has to be four times with anaesthesia*.
  Finally, how much time the treatment is expected to take and how much time is available are also perceived to be essential parts of the treatment planning since they control the extent of the single treatment sessions.
- *Mm, 30* *min where you should go through the pocket probing depth, redebride, approximal cleaning, polishing and then you should also explain to the patient how to keep it clean and where to keep clean and with which tools. It takes a bit more than 30* *min*.

### Constituent B: importance of oral hygiene

Oral hygiene is experienced as an integral part of the periodontal treatment and as a relevant factor when it comes to achieving successful results. The instructions related to a patients’ self-care are perceived to be vital elements when conducting a periodontal treatment.

- *You could see somehow that here was a need to sort of, parallel to the actual treatment that you plan, to clean maybe debridement-wise so one should always have oral hygiene as a foundation. Because it is somehow.., my theory is that oral hygiene is the basis of the whole*.
  This element was expressed in two different ways: in a positive way where a good oral hygiene would lead to positive results or in a negative way expressing that if oral hygiene was not adequate the periodontal status would never improve.
- *Eeh, Well I, showed these other brushes, which may be easier for her. But at the same time I see that it’s actually her job now. I’ve done my.., my main part anyway. I should be there and offer support all the way later on too, but.., but it is her job to every, every day be there and do this. Otherwise it will never be good*.
  Furthermore, it is experienced as being important that the patient understands what impact the oral hygiene has on the periodontal disease and that it has to be successfully incorporated into the daily routines.
- *Then there is this last stretch to make it a routine, that it should be every day. That is the hardest*.
  In addition to making the oral hygiene measure a daily routine it is experienced that patients should also receive clear instructions in which oral hygiene appliances to use and how, in order to avoid possible frustration that might result in the discontinuation of their use.
- *Eeh… It is.., I usually think that the patient must.., They must be able to use what I give them as tools. If it does not work, then you don’t do it and then it is hard.*

However, it is commonly perceived that patients progress in their self-care ability as they usually adhere to the given instructions and keep up their motivation over time, even though this is not always obvious during the active treatment phase.

### Constituent C: self-awareness and motivation of the patient

In the initial part of the periodontal treatment, the patients are experienced as self-aware with regards to their oral condition to a certain degree and partially aware of what is required of them in order to change their habits e.g. regarding their oral hygiene.

- *He.., he sort of knew a little about what the situation was. And it is good that they have a kind of.., this self-awareness that* ‘*Yes, I know this is what it is like, but I might not have handled it quite right*’*. That’s the sort of.., perhaps you could call it a classic statement*.
  On the other hand, these habits could mean different things to different patients and could also involve smoking cessation.
- *She has cut down on the smoking a little bit, but it is really not many cigarettes that she has put out. Eeh, but I also say that.., this work that I do, it is just so important that she quits smoking for us to expect it to heal well. But it doesn’t feel like she is quite there yet, to quit smoking*.
  The last citation could also implicitly mean that it is experienced as vital for patients to gain a better self-awareness of their own situation in order to become more motivated to change their habits. This has also been expressed explicitly by other participants.
- *Mm. That’s when he said he did not want to let go, like, he thinks it feels okay.* ‘*Even though I do not like to come here, it feels better in my mouth, the gums feel healthier*’*. Eeh… So he has experienced a feeling at home himself, that it does not bleed as much when brushing and so. So that’s where I think he has understood it, the context*.

### Constituent D: support and doubt

In the relation to the phenomenon it is experienced as important to be supportive of the patient during the periodontal treatment.

- *It is not my role to be their parent or point fingers, but to just always being guiding and advising and supportive*.
  Moreover, an experience of doubt towards the patient was also apparent in this constituent. This is merely expressed as a doubt of the patient’s adherence and compliance with oral hygiene instructions e.g. a perceived uncertainty regarding whether or not the patient has understood the given information.
- *Yes. So it.. they understand it, but then I do not know if they always hear it. It’s as if you say, they.., you look at them and they nod. And then they leave the room, then I do not know what happens*.

On the other hand, patients sometimes declare that they have complied with the instructions while not showing any objective signs of this compliance. This creates an experience of doubt of the patient, which could in some instances result in a minor frustration.

### Constituent E: mechanical infection control

The mechanical infection control is mostly experienced as being successful, both in its execution and when considering the obtained results following completion of the procedure.

- *Eeh, no, I thought it went pretty smoothly. Sure, there were some sites.., when you get to the molar area like so when you think of yes, furcation areas and so on*.
  In the previous quote, we can clearly see a successful execution of the procedure but also an insinuation that some areas in the oral cavity are experienced as more complicated to treat. This has also been clearly expressed in the participants’ descriptions of where difficulties are experienced when performing scaling in regard to gaining a good visual of an area or accessing a certain area with instruments.
- *Eeeh abundant bleeding, did not see anything, very edematous gingiva, eeeh redness, eeeh… yes, and since there were food leftovers and so on.., it was a lot of.., a lot suctioning, a lot of picking out. It was sort of like food impaction you might say.., between some molars where it like gets stuck… so that it was.. it was difficult*.
  Regarding the instruments used during scaling, the ultrasonic instruments and hand instruments were perceived to complement each other resulting in a more thorough cleaning of the teeth.
- *sometimes, I feel that when you are sitting and scraping by hand, which I think is very nice to do on these patients when there’s a lot, but afterwards when using the ultrasonic so somehow, it sort of bubbles up.., it is like you drag up all the rubbish that partly remains*.

## Discussion

The results from this study have described what characterises the phenomenon of performing a periodontal treatment in the context of general dentistry. In particular, the results have enabled us to obtain a deeper, phenomenological understanding of what it psychologically means to conduct a non-surgical periodontal treatment in the context of general dentistry. The results awaken a curiosity to further understand the reasons behind the treatment strategies in general dentistry and why patients might receive non-individually tailored periodontal treatment.^12,13^

One of the constituents of the phenomenon explored in this study concerned treatment planning (Constituent A) that is perceived as a routine task that follows a standardised workflow accounting for the time available and presence of deep periodontal pockets. This could perhaps indicate that the treatment is not individually tailored to the specific patient. On the other hand, patient-specific characteristics are also evident in the treatment planning e.g. systemic disease, stress management, and smoking habits which are important considerations in the treatment process.^25,26^ It was also experienced that the available treatment time in the appointment register determines the extent of treatment sessions. A deeper understanding of this particular characteristic of the phenomenon was not possible to obtain since the participants did not explain the reason for perceiving these time limits. However, a recent study where a similar issue was explored indicated that DHs in general practice would occasionally experience pressure to give priority to scaling over patient education and a tight schedule was the most common reason for this prioritisation.^19^

Patient education is a two-way relationship between the professional and the patient. This means that the knowledge should not only be conveyed to the patient, but that the patient should also grasp the knowledge to a certain degree so that they themselves experience a need to change a habit. This parameter is important in the treatment of periodontal diseases since good oral hygiene is necessary in order to achieve good results during the active phase of treatment, but also helps the patient achieve healthy oral conditions by preventing the occurrence of periodontal diseases.^6^ This behavioural element is recognised within the explored phenomenon in this study because it was experienced as being important to include oral hygiene instructions in the periodontal treatment (Constituent B). Furthermore, it was also apparent that patients need to understand the benefits of good oral hygiene and incorporate it as a part of their daily routines in order to achieve a successful outcome. This change is not always easy to accomplish. However, it has been previously shown that patients, who understands what measures need to be taken to avoid tooth loss, incorporate the given oral hygiene measures as a daily routine.^27^ The patient’s self-awareness is perceived to be a central part of the periodontal treatment (Constituent C). If patients perceive the seriousness of their disease and understands the benefits of a behavioural change, they could become more motivated to comply with the oral hygiene instructions they receive.^11^ Motivating the patient is one part of the action taken to improve their oral hygiene routines. Another vital part, which is experienced (Constituent B), is to instruct the patient in proper oral hygiene technique and recommend appliances that are individualised to the patient. This is essential since some appliances, like the inter-dental brushes, are somewhat complex to use and require good manual skills as well as commitment from the patient.^28^ If a patient receives accurate and detailed instructions they themselves perceive the self-care to be possible to perform. If they comprehend the self-care and see it as a manageable achievement, their motivation will even be strengthened,^29^ which was also apparent in constituent B. Therefore it could be of vital importance for clinicians to ensure that proper, clear information is given to the patient and that they are sensitive to the patients’ needs in order to enhance the patients’ feeling of control in the situation where the treatment occurs.^30^

With regards to giving proper oral hygiene instructions and motivating the patient, a need to be supportive of the patient was also experienced (Constituent D). A similar matter has been addressed in another study where the DHs considered the establishment of a trusting relationship between themselves and the patient as their responsibility and in doing this a supportive attitude was important.^18^ Furthermore, if the professional clinician and the patient has a good relationship, the patient will be more satisfied with the periodontal care provided.^31^ The role of the clinician could also mean that they become more engaged or concerned with the patient’s oral health by acting as a supporter instead of a supervisor or an expert. In those instances, the patient might perceive a collaborative alliance with the clinician and hence be more motivated to improve their self-care or attain good health.^29^ On the other hand, it is not always the case that patients understand what is actually required by them in order to maintain their oral health, even though they have received the necessary information about their disease from a supportive clinician.^27^ Consequently, the doubt, as present in the lived experience of performing a periodontal treatment (in this particular study), does not come as a surprise. The uncertainty regards the question if the patients understand the information provided by a professional and/or if they actually comply with the instructions provided. This in turn might result in the clinician losing hope, which could influence the clinicians’ motivation to provide the necessary pedagogical attitude required for a successful treatment. However, one has to recognise the presence of an engagement in the lived experience of performing a periodontal treatment by the perceived need to be supportive which shows willingness to provide the best treatment possible.

The treatment itself does not merely consist of communication and interpersonal relations between the clinicians and the patients. Another part of the treatment is the mechanical infection control. Scaling is an effective treatment when considering the goal to reduce inflammation and pocket depth in patients with periodontal diseases.^28^ The last Constituent (E) of the phenomenon related to this treatment modality, where this treatment measure was experienced as successful, is consistent with previous results.^32^ However, difficulties were experienced with gaining visibility and accessing certain teeth, mainly molars. This difficulty has previously been acknowledged, as this treatment modality is less effective in treating molars with furcation involvement. Surgical treatment might improve the treatment outcome as such treatment provides better visual and physical access.^33,34^ Lastly, the combination of ultrasonic and hand instruments was experienced to result in a more thorough cleaning of the teeth although separate use would provide a similar clinical outcome^35^ even in patients with severely advanced periodontitis.^36^ All in all, this means that a proper understanding of the abilities and limitation involved in scaling is a fundamental part of the lived experience of periodontal treatment.

Understanding what it means to perform a non-surgical periodontal treatment does not necessary mean that the rationale behind the performed treatment is unveiled as it was intended in the first place. It is not possible to fully explain why certain clinicians treat periodontally compromised patients in a standardised fashion. On the other hand, factors (constituents) pertinent to the individual patient are affecting the whole *treatment-process-in-action* as well and are accounted for in the description of the phenomenon e.g. how oral hygiene instructions are presented to the patient and which inter-dental brushed are recommended. This, in turn, could give the community a more nuanced picture of the clinician–patient interaction on a deeper level which could help us in several ways—one being when formulating guidelines to clinicians. If these guidelines are based on clinicians’ own experience of conducting a periodontal treatment, they could be easier to implement. In addition, this material could help the research community discover new research questions which could further help us understand the periodontal treatment context as a whole.

### Methodological considerations

One of the important factors for the validity in DPPM is the selection of the participants as we need to have a valid description of a situation where the phenomenon has been experienced. Hence, participants were purposely selected in order to acquire a rich description of a situation where an individual has performed a periodontal treatment.^21^ Consequently, DHs from the general dentistry setting were chosen since they have affirmed experience of performing non-surgical periodontal treatment independently and they themselves could provide an in-depth account of such a situation. By performing this purposive sampling, one could assure that a valid description of a situation, where the phenomenon had appeared, would be obtained. Furthermore, general dental practitioners were not included in this sample since almost all non-surgical periodontal treatment in general dentistry in Sweden is performed by DHs.^17^ By interviewing three participants with rich descriptions and using eidetic variations when analysing the description, one could remove the characteristics of the phenomenon which are pertinent to the particular individual experiencing them. This process makes it possible isolate constituents instead that are general to the phenomenon, hence, reach another level of abstraction. In conclusion, important, patient-related, factors constitute the lived experience of performing a periodontal treatment. However, an experience that the pre-existing standardised workflow influences patient management was also present. This could partly explain why patients might not receive a truly individually tailored treatment. Therefore, it is important for dental care stakeholders to acknowledge this issue and formulate/implement guidelines where periodontal treatment routines are more adaptable to the individual patient and not standardised.
